# Supplementary material for: Is Olfactory Testing a Useful Diagnostic Tool to Identify SARS-CoV-2 Infections Early? A Cross-Sectional and Longitudinal Analysis
Source: J Clin Med. 2023 Apr 27;12(9):3162. doi: 10.3390/jcm12093162 (PMC10179328; doi:10.3390/jcm12093162)
Supplement: Supplementary file 1 [file jcm-12-03162-s001.zip › jcm-2200700-supplementary.pdf]

Journal of
Clinical Medicine

MDPI

SUPPLEMENTARY MATERIALS

Patients

At enrollment, all participants of both study parts completed questionnaires assessing basic demographic, clinical and epidemiological information. In the longitudinal study part, participants, who were tested positive for a SARS-CoV-2 infection, daily evaluated general clinical (such as fever, headache, etc.) and ear, nose and throat (ENT) symptoms since the disease onset in a personnel questionnaire with a five-point scale (0 = no symptoms; 3 = severe symptoms). A parent of legal guardian assisted in completing questionnaires for children <18 years of age.

Severity of the clinical symptoms was assessed subjectively by a questionnaire. Classification into the individual severity levels was carried out in accordance to NIH guidelines in a slightly modified form [1]:

Asymptomatic course of disease: Individuals, who were tested positive for SARS-CoV-2 using a virologic test (i.e., a nucleic acid amplification test [NAAT] or an antigen test) but who have no symptoms that are consistent with COVID-19.

Mild course of disease: Individuals who have any of the various signs and symptoms of COVID-19 (e.g., fever, cough, sore throat, malaise, headache, muscle pain, nausea, vomiting, diarrhea, loss of taste and smell) but who do not have shortness of breath, dyspnea, or abnormal chest imaging.

Moderate course of disease: Individuals who show evidence of lower respiratory disease during clinical assessment or imaging and who have an oxygen saturation measured by pulse oximetry (SpO_2) ≥94% on room air at sea level.

Severe course of disease: Individuals who show evidence of lower respiratory disease (with an oxygen saturation measured by pulse oximetry (SpO_2) ≥94%) and a pronounced expression of further clinical symptoms (fever, cough, sore throat, malaise, headache, muscle pain, nausea, vomiting, diarrhea, loss of taste and smell).

Olfactory Function Assessment

Accordingly, a test was considered positive for relevant OD if at least two test results were incorrect. Severity of OD was determined based on the quantitative test results: grade 0 = five or four correct test results and no OD; grade 1: three correct test results, mild OD; grade 2 = two correct test results, moderate OD; grade 3 = one correct result, severe OD; grade 4 = no correct test result, anosmia. Hyposmia was defined as mild to severe OD (grade 1–3), while anosmia was characterized by complete loss of smell (grade 4). Each card contained a different composition of odors in order to avoid falsification of results due to repetitive performance of the test.

Table S1. Univariate and multivariate analysis of factors associated with olfactory dysfunction (*n* = 494).

	OD (*n* = 226)	Univariate	Multivariate	
		p value	OR (95% CI)	*p* value
Gender, *n* (%)				<0.001
Males	158/307 (51.5)	<0.001	2.44 (1.58–3.77)	
Females	67/187 (35.8)			
Age (years)	67 (16–94)	<0.001	0.95 (0.94–0.97)	0.001
Current smoking, *n* (%)	41/46 (89.1)	0.74		
Comorbidities				
Diabetes mellitus, *n* (%)	52/101 (51.5)	0.21		
Pulmonary diseases, *n* (%)	33/53 (62.3)	0.02		
Cardiovascular diseases, *n* (%)	54 (10–538)	<0.001		
Malignancy, *n* (%)	57/126 (45.2)	0.85	1.65 (0.83–3.28)	0.15
Renal diseases, *n* (%)	33/69 (47.8)	0.74	1.15 (0.74–1.80)	0.53
Hepatological diseases, *n* (%)	21/53 (39.6)	0.20		
Preoperations in the ENT tract, *n* (%)	1/3 (33.3)	0.66		

Abbrevations: ENT, ear nose throat; OD, olfactory disease.

Table S2. Sub-analysis of factors associated with olfactory dysfunction in patients infected with the SARS-CoV-2 Delta variant (*n* = 42).

Characteristics	Olfactory Dysfunction (*n* = 33)	No Olfactory Dysfunction (*n* = 9)	*p* value
Males, *n* (%)	15 (45.5)	7 (77.8)	0.04
Age (years), median (range)	34 (12–59)	36 (12–47)	0.09
Current smoking, *n* (%)	12 (36.4)	0 (0)	0.03
General symptoms			
Fever, *n* (%)	6 (18.2)	0 (0)	0.32
Fatigue, *n* (%)	11 (33.3)	2 (22.2)	1.00
Arthralgia, *n* (%)	4 (12.1)	0 (0)	0.57
ENT			
Sore throat, *n* (%)	7 (21.2)	1 (11.1)	1.00
Nasal obstruction, *n* (%)	16 (48.5)	2 (22.2)	0.43
Rhinorrhea, *n* (%)	16 (48.5)	2 (22.2)	0.69
Pneumological			
Cough, *n* (%)	17 (51.5)	2 (22.2)	0.70
Shortness of breath, *n* (%)	1 (3.0)	0 (0)	1.00
Digestive			1.00
GI symptoms (nausea, vomiting, diarrhea), *n* (%)	0 (0)	1 (0)	
Neurological			
Taste disorders	1 (3.0)	1 (11.1)	0.36
Headache	10 ()	1 (11.1)	0.41

Table S3. Sub-analysis of factors associated with olfactory dysfunction in patients infected with the SARS-CoV-2 Omicron variant (*n* = 48).

Characteristics	Olfactory Dysfunction (*n* = 27)	No Olfactory Dysfunction (*n* = 21)	*p* value
Males, *n* (%)	15 (55.6)	4 (14.2)	0.03
Age (years), median (range)	30 (112–65)	25 (12–58)	0.09
Positive smoking history, *n* (%)	8 (29.6)	2 (9.5)	0.04
General symptoms			
Fever, *n* (%)	2 (7.4)	1 (3.7)	1.00
Fatigue, *n* (%)	7 (25.9)	3 (14.3)	0.49
Myalgia or arthralgia, *n* (%)	7 (25.9)	1 (3.7)	0.12
Loss of appetite, *n* (%)	0 (0)	0 (0)	
ENT			
Sore throat, *n* (%)	5 (18.5)	0 (0)	0.06
Nasal obstruction, *n* (%)	21 (77.8)	11 (52.4)	0.20
Rhinorrhea, *n* (%)	21 (77.8)	11 (52.4)	0.20
Pneumological			
Cough, *n* (%)	16 (59.3)	8 (38.0)	0.23
Shortness of breath, *n* (%)	0 (0)	0 (0)	
Digestive			
GI symptoms, *n* (%)	0 (0)	0 (0)	
Neurological			
Taste disorders, *n* (%)	1 (3.7)	0 (0)	1.00
Headache, *n* (%)	9 (33.3)	7 (4.8)	1.00

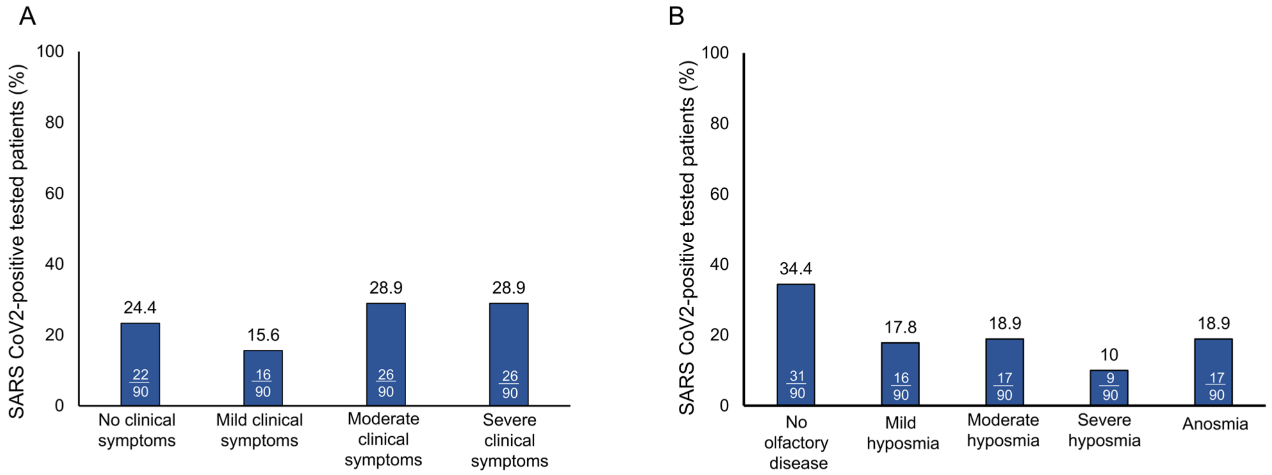

Figure S1. Intensity of clinical symptoms during SARS-CoV-2 infection. Intensity of (**A**) classical clinical symptoms and of (**B**) olfactory dysfunction in SARS-CoV-2 positive tested individuals.

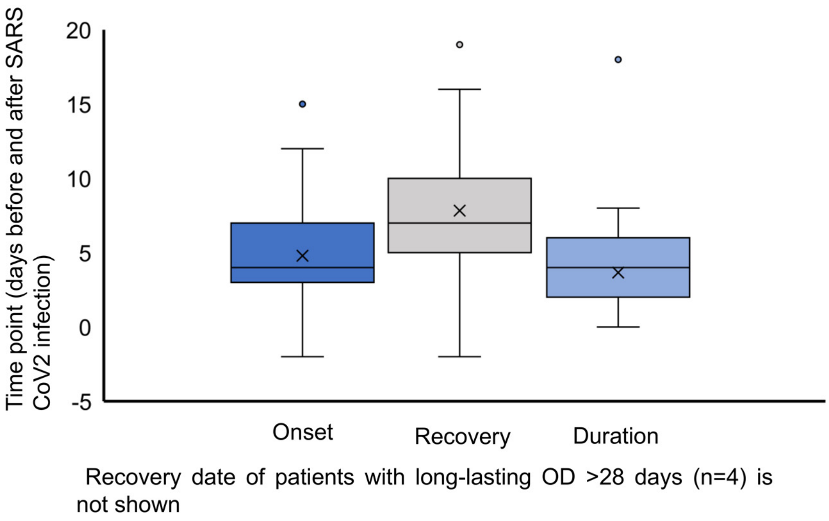

Figure S2. Clinical course of clinical symptoms during SARS-CoV-2 infection. Onset, duration and recovery of olfactory dysfunction in SARS-CoV-2 positive tested individuals.

References

1. Clinical Spectrum. Available online: https://www.covid19treatmentguidelines.nih.gov/overview/clinical-spectrum/ (accessed 26 February 2023).
